# Navigating Fusarium wilt of bananas: a ready-to-use subset of resistant *Musa* genotypes

**DOI:** 10.3389/fpls.2026.1753570

**Published:** 2026-03-30

**Authors:** Mathieu Rouard, Guy Blomme, Walter Ocimati, Julie Sardos, Bonaventure A. Omondi, Ricardo Oliva, Max Ruas, Jorge E. Vargas, Si-Jun Zheng, Bart Panis, Sebastien C. Carpentier

**Affiliations:** 1 Bioversity International, Parc Scientifique Agropolis II, Montpellier, France; 2 Bioversity International, Addis Ababa, Ethiopia; 3 Bioversity International, Kampala, Uganda; 4 Bioversity International, Cotonou, Benin; 5 International Center for Tropical Agriculture (CIAT), Cali, Colombia; 6 Bioversity International, Kunming, Yunnan, China; 7 Yunnan Key Laboratory of Green Prevention and Control of Agricultural Transboundary Pests, Agricultural Environment and Resources Institute, Yunnan Academy of Agricultural Sciences, Kunming, Yunnan, China; 8 Bioversity International, Leuven, Belgium; 9 KULeuven, Laboratory of Tropical Crop Improvement, Leuven, Belgium

**Keywords:** banana, disease resistance, *Fusarium oxysporum* f. sp. cubense, Fusarium wilt, genebanks, *Musa*, seed systems

## Abstract

Fusarium wilt Tropical Race 4 (TR4) poses one of the greatest threats to global banana production, with major implications for food security and sustainable agriculture. Harnessing natural genetic diversity offers a primary line of defense, but progress is hindered by limited access to resistant or tolerant cultivars and the lack of harmonized resources. In this study, we identified and curated a priority subset of 37 TR4-resistant banana accessions from the International *Musa* Germplasm Transit Centre (ITC). These accessions were enriched with agronomic and genetic information and prioritized through a conservation and distribution framework at ITC to ensure their availability. This resource provides certified, diverse and well-documented planting material and ensures availability for breeders, researchers, and farmers. By strengthening global seed systems and linking to community-level multiplication, it can enable the distribution of TR4-resilient bananas in diverse agroecological contexts. This curated collection represents a critical step toward sustainable solutions for managing TR4 worldwide.

## 1 Introduction

Bananas (*Musa* spp.) are a popular staple food in many tropical and subtropical countries, helping ensure food and nutritional security for hundreds of millions of people. More than 100 countries annually produce around 180 million tons (Food and Agriculture Organization of the United Nations, 2023), comprising 69% bananas produced used as dessert, and 31% bananas for cooking, with exports relying predominantly on clones from the (dessert) Cavendish cultivar group (Dawson et al., 2024). Global banana production, particularly but not exclusively the Cavendish group, is endangered by *Fusarium oxysporum* f. sp. *cubense* Tropical Race 4 (Foc TR4), a highly virulent strain of the soil-borne Fusarium wilt fungus (FAO, 2024). TR4 has now been confirmed in 23 countries, mainly across South and Southeast Asia, but also in Africa, Latin America, the Middle East, and Oceania. The first outbreak in Latin America was reported in Colombia in August 2019 (García-Bastidas et al., 2020), followed by Peru in April 2021 (Acuña et al., 2021), Venezuela in January 2023 (Mejías Herrera et al., 2023) and Ecuador late 2025 (Press release). Importantly, there are major economic and food security risks posed by Foc TR4, particularly in already affected countries such as China, Colombia, Laos, Peru, the Philippines, and Vietnam (Ritter et al., 2024, Ritter et al., 2025). Because the pathogen persists in soil and chemical control options are limited, the identification and deployment of resistant genotypes and their interaction with the microbiome remain one of the most promising long-term strategies for sustainable production (Liu, 2025).

Fusarium wilt TR4 is not solely a problem for Cavendish bananas. Several studies have investigated the response of various banana cultivars to Foc TR4 (reviewed in Munhoz et al., 2024). Several wild species show resistance or lower levels of susceptibility (Li et al., 2015). Cavendish, Pome, Silk and Gros Michel are all significantly important cultivars (internationally or locally) that are highly susceptible to the pathogen. However, several cultivars from the groups Iholena, Ney Mannan, Plantain, and Mutika/Lujugira are also important in local markets, and have been found TR4-resistant (Rocha et al., 2021; Munhoz et al., 2024; Thangavelu et al., 2025). Notably, resistance can also vary between closely related cultivars within the same clonal groups, highlighting the importance of comparative evaluation using well-defined and traceable plant material.

Recent systematic reviews have already summarized TR4 resistance sources across diversity of cultivated bananas, including an overview of reported resistant genotypes across studies (Rocha et al., 2021; Munhoz et al., 2024). However, translating published resistance reports into subsequent research actions, breeding, and germplasm dissemination depends critically on access to the exact genetic resources that were evaluated. Confidence in their identity and traceability is therefore of prime importance. In banana, ambiguity in cultivar naming (e.g homonyms or synonyms) and clonal variation can complicate comparisons among studies, while international exchanges of planting material is often constrained by phytosanitary requirements and legal frameworks. As a result, materials described in TR4 screening studies are not always readily accessible for follow-up work or broader testing, despite their potential value.

International Genebanks play a pivotal role in preserving and making accessible to users the global diversity of major crops and trees germplasm. The International Musa Germplasm Transit Centre in Leuven, Belgium (ITC) is the world’s largest and most diverse collection for banana genetic resources. It conserves and distributes, widely, for free or at cost-recovery virus-free, disease-indexed, true-to-type accessions under the Standard Material Transfer Agreement (SMTA), thus enabling reproducible evaluation, breeding, and equitable germplasm sharing (Van Den Houwe et al., 2020). However, and despite a broad range of users including national programs and the research community, tapping into this wide diversity (>1700 accessions) is not always easy as selection criteria and the above-mentioned problems on traceability and identity can be difficult to deal with. In this context, the availability of subsets tailored to specific traits can considerably ease germplasm selection as well as providing a standardized framework for evaluation, use and trait development.

This paper aims to: (i) critically review and synthesize TR4 screening studies using ITC-distributed material; (ii) analyze methodological heterogeneity across field and controlled evaluations; (iii) define a curated subset of Musa accessions available in ITC with consistent evidence of TR4 resistance and a resistance score; and (iv) discuss possible use of this subset and pathways for integrating these accessions into banana seed systems.

## 2 Materials and methods

### 2.1 Literature review and curation for Foc TR4 resistance

We conducted a structured literature search to identify studies assessing resistance to *Fusarium oxysporum f.* sp. *cubense Tropical Race 4* (Foc TR4) in Musa genotypes sourced in the International Musa Germplasm Transit Centre (ITC). We screened peer-reviewed publications and grey literature (theses, technical manuals/guidelines, book chapters, and conference proceedings) published in English. Searches were performed in Europe PMC (https://europepmc.org/advancesearch) , Google Scholar labs (https://scholar.google.fr/scholar_labs) using sentence such as ‘list of studies assessing fusarium wilt resistance TR4 in musa banana genotypes from the International transit center (ITC) leuven’, and CGSpace (https://cgspace.cgiar.org) as of February 2026. In Europe PMC, we used an advanced query combining “banana”, “Fusarium wilt” and “tropical race 4” with ITC related terms in funding, acknowledgements or methods fields. We complemented this with targeted searches in Google Scholar and CGSpace to capture relevant documents not indexed in Europe PMC. Records were retained when they reported phenotyping for TR4 resistance and explicitly indicated the use of ITC-sourced material. False positives were discarded when they did not address TR4 resistance (e.g., other traits such as drought tolerance, other Foc races), or when they were primarily focused on genetics without generating TR4 phenotyping and relied on other studies for resistance classification. When milestone outputs were later replaced by peer-reviewed papers reporting the final results, we retained the final publication only to avoid duplicates. Finally, two additional expert-identified resources were included: the first one was published in Chinese and not available in English and the second one used ITC accessions without using explicit references in the public text (**Supplementary Table 2**).

The passport data of these accessions was retrieved from the Musa Germplasm Information System (MGIS) (Ruas et al., 2017), complemented by a new source of information unraveled by several molecular approaches clarifying taxonomic classifications (Martin et al., 2023; Sardos et al., 2025). We prioritized Foc TR4 screening studies that included field evaluations, or both field and greenhouse/pot evaluations. Studies based solely on pot experiments were retained only when they provided supplementary results for accessions already assessed in field trials in other studies. Finally, we incorporated predicted Foc TR4 responses derived from marker allele profiles (Ferreira et al., 2024) for those accessions in our sample for which evaluation data was available.

### 2.2 Method and criteria for subset selection

To identify a subset of banana accessions for further investigation into Foc TR4 resistance, we applied a stepwise selection process. First, we included accessions that were rated as ‘immune’, ‘highly resistant’, or ‘resistant’ in published evaluation studies. All chosen accessions are available for distribution through the ITC banana genebank, where their genetic identity and integrity have been verified. We considered “resistance” as reported in each study, acknowledging that this may reflect a continuum from true immunity to field-level tolerance depending on the scoring method used. Second, to maximize genetic diversity, we selected both wild Musa species and cultivated types, covering cultivars used as dessert and cooking. Thus, we gave priority to accessions consistently classified as resistant in multiple studies, or those supported by additional datasets such as genomic sequences, trait-value data, or male fertility reports (Mukasa and Rubaihayo, 1993; Dumpe and Ortiz, 1996; Rosales et al., 1999). Where available, complementary information on resistance to other major pests and diseases, abiotic stress tolerance, nutritional properties, and agronomic or sensory traits was also considered. Finally, the preliminary list was refined and validated through consensus among the co-authors, who brought expertise in germplasm conservation, genomics, phenotyping, phytopathology, and germplasm conservation from Africa, Asia, Europe, and Latin America. The complete list is provided in **Supplementary Table 1** and includes known susceptible accessions, such as Cavendish, which serve as reference checks.

### 2.3 Ensuring plant material availability

When managing a clonal, vegetatively propagated collection such as banana, a central priority is ensuring a reliable supply of plantlets for distribution. Because bananas are largely sterile and propagated through vegetative means, field maintained material quickly accumulates pathogens and clonal variation over time, which makes *in vitro* conservation critical to preserve genetic integrity and phytosanitary status. Also, in an *in vitro* collection, subculture frequency can vary up to tenfold depending on genotype, cultivar, or genomic group (Van Den Houwe et al., 2020). To address this, the ITC genebank applies protocols that track plant requests and adjust regeneration schedules accordingly, to enable timely anticipation of demand. The TR4-focused subset highlighted in this study has now been prioritized within these schedules, ensuring that sufficient material is proactively maintained. Distribution is carried out under SMTA and strict phytosanitary protocols, guaranteeing legal compliance and certification to international and national standards. This reduces regulatory delays and provides researchers and breeders with rapid, secure access to high-quality planting material.

## 3 Results

### 3.1 Perspective from the literature

Based on the literature review, we retrieved a total of 201 records (Europe PMC, n=56; Google Scholar, n=50; CGSpace, n=93; expert-identified resources, n=2) and screened them for studies evaluating Tropical Race 4 (TR4) resistance in banana accessions from the International Musa Germplasm Transit Centre in Leuven, Belgium (ITC). After excluding false positives (e.g., records focusing on other traits or other Fusarium races, genetics-only studies without TR4 phenotyping, or studies that did not use ITC-sourced material), we retained 11 scientific studies published between January 2014 and September 2025. These studies reported TR4 phenotyping of ITC-distributed material and collectively described 170 distinct genebank accessions. This represented about 10% of the complete genebank collection, with some accessions appearing in multiple studies (**Supplementary Table 1**). Five studies were conducted under both pot/screenhouse and field conditions (Zhang et al., 2018; Zuo et al., 2018; Dita et al., 2020; Zhan et al., 2022; Thangavelu et al., 2025), allowing for direct comparison of results. Three others were carried out exclusively in greenhouses (Chen et al., 2019; García-Bastidas, 2019; Liu et al., 2021), while three were limited to field trials, sometimes in different countries (Molina et al., 2016; Gueco et al., 2017; Mintoff et al., 2021). We conducted a comprehensive review of the inoculation and screening methods used across all papers. The analysis revealed notable methodological differences, particularly in pot and screenhouse trials. While some studies provided detailed descriptions of their methods, others lacked sufficient details for further comparison. Only 2 studies used the same inoculation method, while differences in sampling, environmental conditions, and diverse soil types across various regions worldwide could collectively contribute to variations in the reported results (**Supplementary Table 2**).

For instance, significant variation in resistance expression between controlled (screenhouse) and field conditions has been reported. In China, 42.1% of genotypes became more resistant in field trials, while 21.1% remained unchanged and 36.8% became more susceptible (Zhan et al., 2022). Following a similar trend, in India, 41.9% increased resistance in the field, 33.3% were unchanged, and 24.7% decreased (Thangavelu et al., 2025).

Moreover, substantial variation in TR4 resistance was also reported within the same cultivar group under the same field conditions. For example, most Iholena accessions are classified as susceptible, yet some, such as Kofi (ITC0912), exhibit resistance. Similarly, two Maia Maoli/Popoulu accessions displayed contrasting reactions, with one being highly resistant and another showing extreme susceptibility, and Plantain accessions were mostly resistant but with notable exceptions (Zhan et al., 2022). A well-known example is the giant Cavendish tissue-culture variant (GCTCV) clones obtained in Taiwan Banana Research Institute. These are somaclonal variants of Cavendish that have shown partial resistance or tolerance to TR4 in the field (Hou et al., 2022).

At times, results vary for the same genebank accession. For example, an accession identified as resistant in one study may be classified as susceptible in another. This is exemplified by the Plantain ‘Obubit Ntanga green mutant’ (ITC0519), which was reported as resistant in the Philippines (Molina et al., 2016), highly resistant in China (Zhan et al., 2022) but was recently found susceptible in Indian field trials (Thangavelu et al., 2025), underscoring likely Genotype × Environment × Pathogen interactions.

Finally, regarding marker-assisted selection for TR4 (Ferreira et al., 2024), the marker could predict TR4 resistance in only 3 out of 12 accessions (16.7%) and discrepancies between molecular predictions and field evaluations are observed for nearly all accessions except ‘Pelipita’ (ITC0951) that was evaluated susceptible in greenhouse and predicted susceptible (**Supplementary Table 1**).

### 3.2 Selection of the panel

Among the screened accessions in literature available in ITC, 80% have been assessed for genetic integrity following field verification (Chase et al., 2014) for which 85% were validated. After curating the list of accessions, we selected a subset of 37 genotypes composed of 9 wild accessions (**Table 1**) and 28 cultivated varieties (**Table 2**) that were evaluated as TR4 resistant. All of these accessions are available through the ITC, where they have now been prioritized for distribution.

**Table 1:** Subset of wild banana accessions and their resistance against *Fusarium oxysporum f.* sp. *cubense*, Tropical Race 4.

Accession code	Accession name	Species	Sub species	Country of origin	TR4 reaction	Reference(s)
ITC0249	Calcutta 4	acuminata	burmannica	Myanmar	HR/R	(Zuo et al., 2018; Chen et al., 2019)
ITC0341	Banksii	acuminata	banksii	Unknown	R	(Zuo et al., 2018)
ITC0415	Pisang Cici Alas	acuminata	zebrina	Indonesia	R	(Thangavelu et al., 2025)
ITC0609	Pahang	acuminata	malaccensis	Malaysia	HR/R	(Gueco et al., 2017; Zhang et al., 2018; Zuo et al., 2018; Chen et al., 2019; García-Bastidas, 2019; Liu et al., 2021)
ITC0672	Pa (Rayong)	acuminata	siamea	Thailand	HR/R	(Gueco et al., 2017; Zuo et al., 2018; García-Bastidas, 2019)
ITC0728	Maia Oa	acuminata	zebrina	Martinique	HR	(Zuo et al., 2018)
ITC1120	Tani	balbisiana	–	Thailand	R/MR/S	(Zuo et al., 2018; García-Bastidas, 2019; Thangavelu et al., 2025)
ITC1587	Pisang Klutuk Wulung	balbisiana	–	Indonesia	R	(Zuo et al., 2018)
ITC1526	Musa itinerans	itinerans	–	China	HR	(Zuo et al., 2018)

HR, Highly Resistant; R, Resistant; TR4 reactions correspond to labels provided in respective publications.

**Table 2:** Subset of cultivated banana accessions and their resistance against F*usarium oxysporum f.* sp. *cubense*, Tropical Race 4.

Accession code	Accession name	Genome constitution	Subgroup/variety/cultivar group	Country of origin	TR4 reaction	Reference(s)
ITC0532	Khai (Kampengpeth)	AA	Sucrier	Thailand	R	(Zuo et al., 2018)
ITC0533	Kluai Lep Mu Nang	AA	–	Thailand	HR	(Thangavelu et al., 2025)
ITC0610	Tuu Gia	AA	–	Vietnam	HR	(Zuo et al., 2018)
ITC0611	Pisang Berlin	AA	–	Indonesia	R/S	(Zuo et al., 2018; García-Bastidas, 2019; Thangavelu et al., 2025)
ITC0712	AAcv Rose	AA	–	Indonesia	HR/R	(Gueco et al., 2017; García-Bastidas, 2019; Dita et al., 2020; Thangavelu et al., 2025)
ITC1121	Pisang Lilin	AA	–	Unknown	HR/MR	(Zuo et al., 2018; Thangavelu et al., 2025)
ITC0312	Pisang Jari Buaya	AA	Pisang Jari Buaya	Malaysia	HR/R/S	(Gueco et al., 2017; García-Bastidas, 2019; Thangavelu et al., 2025)
ITC0090	Tjau Lagada	AA	–	Indonesia	R	(García-Bastidas, 2019; Thangavelu et al., 2025)
ITC0662	Khai Thong Ruang	AAA	Ibota	Thailand	R	(Gueco et al., 2017)
ITC0081	Igitsiri (Intuntu)	AAA	Mutika/Lujugira	Burundi	HR/R/MR	(Molina et al., 2016; Gueco et al., 2017; García-Bastidas, 2019; Liu et al., 2021; Thangavelu et al., 2025)
ITC0179	Inkira	AAA	Mutika/Lujugira	Unknown	R	(García-Bastidas, 2019; Liu et al., 2021)
ITC1287	Pisang Berangan	AAA	–	Malaysia	HR	(Thangavelu et al., 2025)
ITC0277	Leite	AAA	Rio	Unknown	R/S	(García-Bastidas, 2019; Thangavelu et al., 2025)
ITC0004	Pisang Nangka	AAA-AAB	Pisang Nangka	Malaysia	R	(Thangavelu et al., 2025)
ITC0127	Kamaramasenge	AAB	Kamaramasenge	Unknown	HR	(Zuo et al., 2018)
ITC0109	Obino l’Ewai	AAB	Plantain	Nigeria	R/MR	(Gueco et al., 2017; Zuo et al., 2018)
ITC0217	Akpakpak	AAB	Plantain	Nigeria	I/HR/R	(Molina et al., 2016; Zuo et al., 2018; Thangavelu et al., 2025)
ITC1668	French Sombre	AAB	Plantain	Cameroon	HR	(Zhan et al., 2022)
ITC0831	Rukumamb	AAB	Iholena-like	Papua New Guinea	R/MR	(Zuo et al., 2018; Zhan et al., 2022)
ITC0912	Kofi	AAB	Iholena	Papua New Guinea	HR	(Zhan et al., 2022)
ITC0649	Foconah	AAB	Pome	Unknown	R/MR/S	(Zuo et al., 2018; García-Bastidas, 2019; Thangavelu et al., 2025)
ITC1327	Poingo	AAB	Maia Maoli/Popoulu	Unknown	HR/R	(Zuo et al., 2018; Zhan et al., 2022)
ITC0338	Blue Torres Strait Island	ABB	Ney Mannan	Australia	R	(Thangavelu et al., 2025)
ITC0659	Namwa Khom	ABB	Pisang Awak	Thailand	R	(Thangavelu et al., 2025)
ITC0472	Pelipita	ABB	Pelipita	Philippines	HR	(Gueco et al., 2017)
ITC0504	FHIA-01	AAAB	hybrid	Honduras	R	(Chen et al., 2019; Mintoff et al., 2021)
ITC0505	FHIA-02	AAAA	hybrid	Honduras	HR/R	(Chen et al., 2019; Mintoff et al., 2021)
ITC1418	FHIA-25	AAB	hybrid	Honduras	HR/R	(Zuo et al., 2018; Chen et al., 2019; Mintoff et al., 2021)

I, Immune; HR, Highly Resistant; R, Resistant; MR, Moderately Resistant; S, Susceptible as reported in their respective studies.

We investigated a broad range of wild accessions spanning several species such as *Musa acuminata*, *M. balbisiana* and *M. itinerans*. Regarding *M. acuminata*, several subspecies are represented with *malaccensis*, *zebrina*, *banksii*, *burmannica* and *siamea*. Across multi−location datasets, *M. acuminata* ssp. *malaccensis* ‘Pahang’, *M. acuminata* ssp. *burmannica* ‘Calcutta 4’, and *Musa itinerans* repeatedly scored in the highly resistant class and remained uninfected in field trials and thus can serve as reference resistant checks. ‘Pahang’ was extensively used in TR4 evaluation studies to understand the physiological responses associated with resistance and including underlying molecular mechanisms (Dita et al., 2011; Zuo et al., 2018; Chen et al., 2019; Zhang et al., 2019).

The investigation panel of 28 cultivated banana accessions reflects substantial genomic and group diversity. The most present genome constitution are AAB (27.6%) and AA (27.6%), followed by AAA (17.2%), ABB (10.35%). Plantain (AAB) is the most frequent, with three accessions, followed by Mutika/Lujugira (AAA) with two. These two clonal groups are more represented in the set due to their importance and intragroup phenotypic diversity. A wide range of additional groups are represented by single accessions, including Pisang Nangka, Iholena, Pisang Awak, Ibota, and Pome. Hybrid varieties originating from breeding programs, including AAAA and AAAB genotypes developed by the Fundación Hondureña de Investigación Agrícola (FHIA) in Honduras, are also represented. Overall, the investigation set includes a mix of bananas that can be used as dessert, cooking and beer types. These accessions reflect a broad geographic diversity, originating from 10 countries across Asia (China, India, Indonesia, Malaysia, the Philippines, and Thailand), Africa (Burundi and Nigeria), Latin America (Honduras), and Oceania (Papua New Guinea). However, 42% of the accessions have an unknown country of origin (**Supplementary Table 1**).

**Figure 1:**
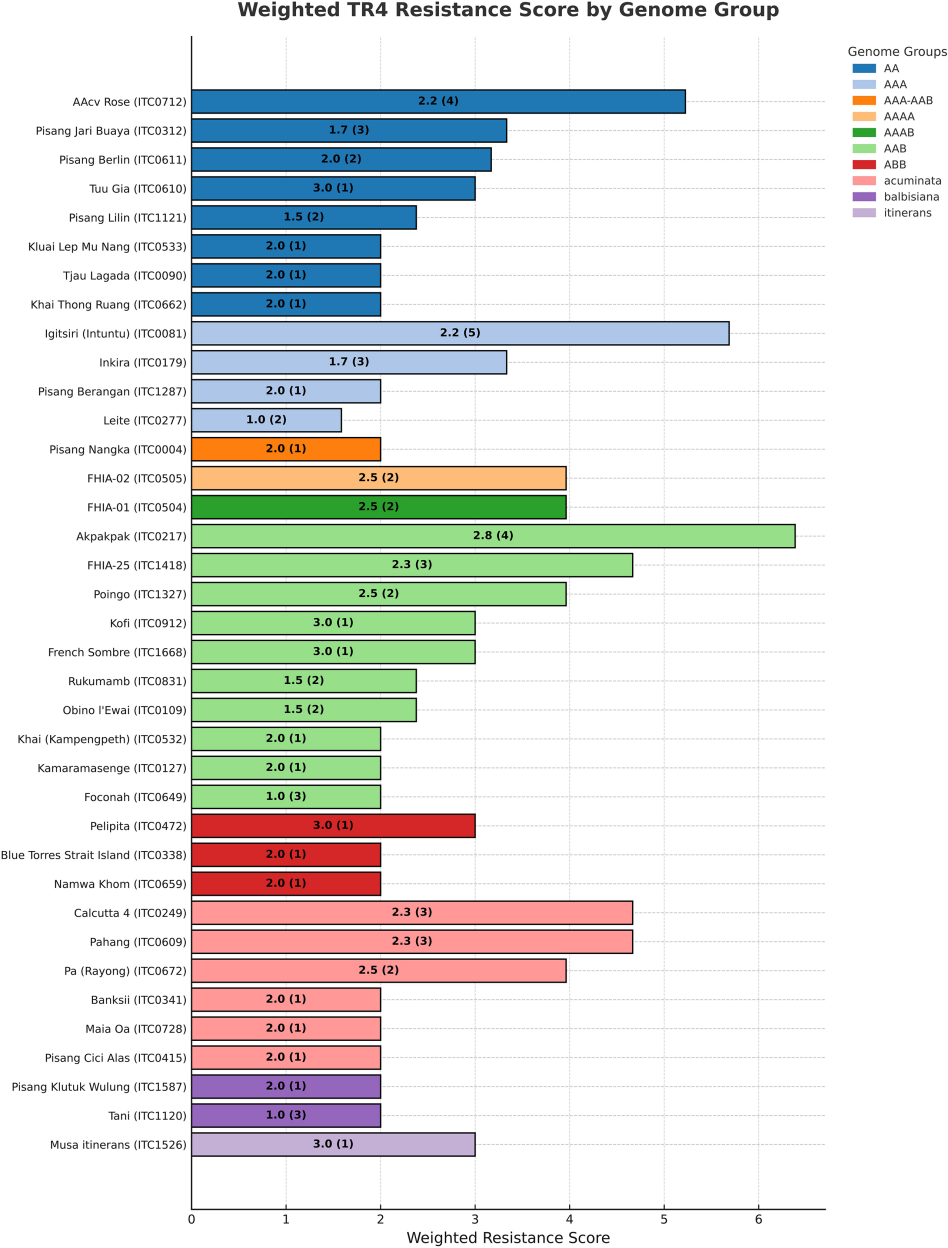
Accession-level TR4 resistance scores for the 37 accessions of the subset, calculated from average resistance ratings (Immune (I)=3, highly resistant (HR)=3, resistant (R)=2, moderately resistant (MR)=1, susceptible (S)=0) and adjusted by the number of studies [AvgScore × log_2_​(Number of studies+1)]. Labels show Average Score and study count between parentheses. Colors indicate the genome group or subspecies classification of each accession. These groups reflect differences in genomic composition (e.g., AA, AAA, AAB, ABB) or taxonomic origin (e.g., *Musa acuminata, M. balbisiana, M. itinerans*).

**Figure 2:**
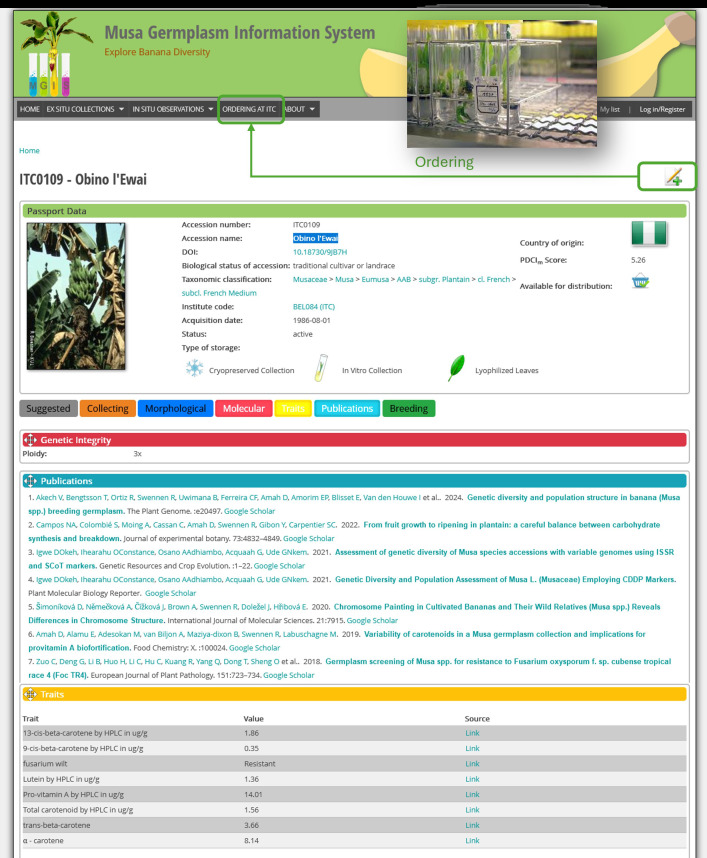
Screenshot of the Musa Germplasm Information System (MGIS) accession page for Obino l’Ewai (ITC0109), illustrating key features including passport data, trait values, associated publications, and material availability for distribution (*in vitro*, cryopreserved, lyophilized). The top navigation highlights the ordering system for requesting germplasm. Color-coded tabs provide access to morphological, molecular, trait, and breeding data, with integrated links to peer-reviewed studies relevant to the accession.

**Figure 1** shows a weighted resistance score per accession, reflecting the average response and number of independent evaluations. All accessions are candidates for resistance, but scores >2 indicate higher confidence, from repeated confirmation of resistance. To facilitate the ordering of this germplasm subset, the corresponding subset was created in MGIS (https://www.crop-diversity.org/mgis/content/ready-use-tr4-resilient-subset), from which plants can be requested (**Figure 2**). Furthermore, each accession is associated with corresponding publications for TR4 evaluation but also for other genotyping or phenotyping studies (Ruas et al., 2017).

## 4 Discussion

Our study targeted *Musa* accessions available from the ITC genebank in Leuven, Belgium with documented TR4 resistance. Some of these accessions have already been well-characterized for other important agronomic traits, fruit quality, drought tolerance, or adaptability to specific growing conditions, while other accessions are lesser well characterized. Identifying a robust set of accessions resistant to *Fusarium* wilt tropical race 4 (TR4) remains challenging because the same accession can display markedly different resistance responses depending on experimental conditions and across independent studies. Plant-pathogen interactions are inherently complex, and resistance phenotypes emerge from the interplay between host genotype, pathogen genotype, other microorganisms present in the rhizome or as endophytes and the environment. Environmental variables such as temperature, humidity, soil moisture, pH, nutrient availability, light intensity, and photoperiod can directly and indirectly influence disease development (Pokhrel, 2021). Environmental factors may increase plant susceptibility through abiotic stress or enhance pathogen growth and virulence, thereby modifying infection pressure. Such complex interactions can explain some of the behavior shifts from resistant to moderately resistant, or vice versa, observed in different studies.

Importantly, most evaluations reviewed here employed different *Fusarium* inoculum sources (**Supplementary Table 2**), introducing additional variability related to strain-specific virulence (Zhang et al., 2024). Variation in inoculum concentration, infection methods, and pathogen genetic background can substantially influence disease outcomes. Plant developmental stage represents another critical parameter. Greenhouse assays frequently rely on young plants grown in simplified substrates under high inoculum pressure, conditions that accelerate symptom development but only partially reflect field realities. While these assays are valuable for standardized and rapid early-stage screening, they should not be considered definitive proxies for long-term field resistance. Field-based validation under agronomically relevant conditions remains essential to confirm durability and stability of resistance.

Heterogeneity observed among accessions within the same cultivar group further complicates interpretation. In banana, cultivar groups generally consist of clonally diversified phenotypes derived from vegetative propagation. However, recent high-resolution genomic analyses have revealed that certain groups, including Pisang Awak and Maia Maoli Popoulu, comprise multiple related yet genetically distinct genotypes (Sardos et al., 2025). Such hidden genetic diversity in studies not working with ITC accessions likely contributes to the contrasting resistance phenotypes reported within cultivars groups. Moreover, genetic and epigenetic variation can accumulate during repeated vegetative propagation or arise during *in vitro* multiplication (somaclonal variation), occasionally altering disease responses. The Giant Cavendish tissue-culture variants (GCTCV) developed at the Taiwan Banana Research Institute exemplified this phenomenon. These somaclonal variants of Cavendish exhibit partial resistance or tolerance to TR4 in the field, resulting from chromosomal deletions and associated gene copy number variations affecting susceptibility loci (Hou et al., 2022; Singh et al., 2025).

At the molecular level, predictive marker approaches provide a valuable framework for large-scale pre-screening. However, reliance on a limited set of markers is insufficient to capture the full spectrum of TR4 resistance present in cultivated bananas (Ferreira et al., 2024). The domesticated banana gene pool is derived from at least 11 ancestral contributors (Carreel et al., 2002; Martin et al., 2023; Sardos et al., 2025), and has a complex mosaic genomic structure. The discrepancies observed between molecular predictions and field evaluations likely arise from multiple, partially independent resistance sources that segregated within this diverse genetic background. Because resistance is ultimately expressed as a physiological phenotype, it may result from multiple small-effect genetic changes that alter plant physiological properties and defense responses. Accordingly, resistance may involve distinct loci, structural variants, gene dosage effects, or quantitative trait architectures that are not fully captured by currently available markers.

Overall, such discrepancies highlight the complexity and challenges of interpreting results across different studies when selecting accessions for resistance. This challenge is intensified when researchers do not have direct access to the genotypes evaluated in each study and must rely on extrapolation based on synonym names or cultivar group classifications.

### 4.1 Why use this panel of banana plants from a genebank?

Using accessions from the International *Musa* Germplasm Transit Centre (ITC) offers several decisive advantages. First, traceability is guaranteed: each accession has a well-documented origin, and researchers worldwide can access the same material again in the future. Second, reproducibility and interoperability are enhanced: because multiple studies can use the same reference accessions, data can be directly compared and integrated across projects, creating a stronger evidence base. Third, genetic precision is ensured: although edible bananas are sterile, clonal diversity arises through accumulated mutations and epigenetic regulation (Jankowicz-Cieslak et al., 2012; Kitavi et al., 2020). This can lead to divergent disease reactions even within a cultivar group. For example, accessions within Iholena and Maia Maoli/Popoulu groups show contrasting responses to TR4, from highly susceptible to highly resistant (Zhan et al., 2022). Such variability underscores the importance of working with accurately identified and stable genebank material, rather than extrapolating from uncertain local sources.

### 4.2 Research applications of TR4-resistant banana accessions

This subset of TR4-resistant banana plants holds significant value for research and a broader understanding of the different mechanisms involved in TR4 resistance.

#### 4.2.1 Evaluation for traits including TR4 resistance

To reliably assess such sources of resistance, a standardized phenotyping protocol was developed (Dita et al., 2020), which enables scientists to evaluate host reactions to Fusarium under both greenhouse and field conditions. By using a common methodology, breeding programs can more accurately compare genotypes and understand how resistance behaves across different environments. It is important to emphasize that the referenced studies were conducted in Asia (China, India, and the Philippines) and Oceania (Australia), under soil and climatic conditions that differ markedly from those in regions such as Africa and Latin America and the Caribbean. To ensure regionally relevant insights, similar evaluations should be conducted in newly Foc-TR4-affected areas of Colombia, Ecuador, Peru, and/or Venezuela. For example, plantain cultivation in Latin America and the Caribbean is increasingly important, both socio-economically and for food security, and accessions from this group must be rigorously assessed for their response to Foc TR4 (Munhoz et al., 2024). As such, prioritizing targeted banana groups in resistance screening is essential, especially since diversity in reactions have been reported in the Plantain cultivar group for example (Zhan et al., 2022; Thangavelu et al., 2025).

An additional challenge is comparing results obtained between the ITC-sourced accessions and local banana and plantain cultivars or clones. After decades of adaptation to different producing regions and selection, local cultivars are now commonly used materials among farmers, but they are also likely to have undergone somaclonal mutation (Heslop-Harrison and Schwarzacher, 2007; Karamura et al., 2010). This highlights the need to i) characterize the genetic identity of local cultivars, ii) determine whether they exhibit such clonal variation, and iii) discuss what types of accessions available in genebanks such as the ITC, or local clones and varieties, should be prioritized for TR4 resistance/susceptibility field studies. Again, the plantain subgroup (AAB) appears particularly relevant, given its wide clonal diversity. These different Plantain cultivars include 79 accessions of the French type (e.g., Dominico), 50 of the False Horn type, and 14 of the True Horn type, as reported by Ibobondji et al., 2023. This diversity is widely distributed across different producing regions, making it a strategic group for evaluating differential responses to TR4.

Moreover, it is important to assess TR4 resistance durability across multiple cropping cycles, as resistance to fusarium can diminish over time due to factors such as pathogen adaptation, environmental variability, and a combination of biotic and abiotic stresses (Khudhair et al., 2014). It is also essential to consider the pathogen inoculum amount/pressure present in the evaluation area. It is also important to establish the minimum number of evaluation cycles that would allow for a more accurate determination of the genotypes’ TR4 resistance or susceptibility. Long-term field evaluations are therefore necessary to determine the stability and robustness of resistance under real-world conditions.

Finally, having such a subset in the field also presents an opportunity to explore additional valuable traits, such as agronomic performance, post-harvest quality, resistance to other abiotic stresses and biotic factors, and nutritional value that would have been underexplored until now. For instance, within the Ney Mannan cultivar group, *Blue Torres Strait Island* exhibits a soft texture and high sugar content, alongside tolerance to cold and good performance on calcareous soils, traits that make it a promising candidate for farmer adoption in diverse environments (Ayala-Silva et al., 2009).

#### 4.2.2 Molecular studies behind resistance: diversity of resistance mechanism

Resistance to Fusarium wilt in banana (*Musa* spp.) follows a plant–microbe interaction model, governed by multiple layers of immune response and controlled by diverse molecular mechanisms (Das et al., 2024; Costa et al., 2025; Yu et al., 2025). Current key strategies include the overexpression of resistance gene analogues (RGAs) (Dale et al., 2017), and the knockout of susceptibility (S) genes, which restrict pathogen colonization (Li et al., 2025). (Xie et al., 2024) Reported that a TR4-resistant Plantain shows an earlier and broader transcriptional response to Foc TR4 than a susceptible Silk cultivar. Despite both being AAB, genes have a different allele dosage effect since both AABs have a different mosaic (Sardos et al., 2025). The subgenome B can be a rich source of defense-related genes associated to Foc TR4 resistance such as resistance-associated gene families (NLR, WRKY22, and LRR-RLK (Xie et al., 2024)). Likewise, understanding the root system’s response to TR4 infection is crucial with evidence for strong responses in internal tissues such as the pericycle, while primary barrier functions are also associated with outer root layers (e.g., exodermis) via lignification and wall reinforcement (Li et al., 2026). It has been shown that the MaKAN4 gene regulates key resistance mechanisms through a zinc (Zn²^+^)-dependent module (MaACA7/MaADH3), providing new foundations for developing disease-tolerant banana cultivars (Huo et al., 2025). Hormonal signaling pathways involving ethylene, jasmonic acid, and salicylic acid also coordinate immune responses to vascular pathogens. This in turn triggers downstream expression of pathogenesis-related (PR) proteins, such as chitinases and β-1,3-glucanases, that inhibit fungal spread (Zeng et al., 2024). Also, structural defenses such as cell wall reinforcement and lignification further hinder pathogen invasion and vascular colonization (Van den Berg et al., 2007).

This diversity of defense mechanisms is partially reflected in the broad genetic variation within banana germplasm, including diploid and wild accessions that exhibit differing TR4 resistance levels. Previous studies such as those reviewed by Rocha et al., 2021 have focused mainly on gene expression in a limited number of genotypes, primarily Cavendish and *M. acuminata* ssp. *malaccensis* ‘Pahang’. Notably, Resistance Genes Analogs (RGA) clusters have been identified in *M. a. ssp. malaccensis ‘Pahang’* and linked to TR4-resistance loci on chromosomes 3 and 10 (Ahmad et al., 2020; Chen et al., 2023). Crucially, the gene RGA2 (Ma03_g09130), isolated from the same subspecies confers TR4 resistance when overexpressed in transgenic Cavendish bananas (Dale et al., 2017). Following the same interaction model, Foc TR4 also relies on its own genetic diversity to adapt to resistant host genotypes. From the pathogen population genomic side, Foc TR4 virulence is supported by genomic plasticity in accessory regions located at the ends of core chromosomes. These regions harbor virulence-associated genes, including the accessory effector SIX4, and show presence/absence variation of accessory gene clusters across race 4 strains (Zhang et al., 2024). Moreover, TR4 strains can carry a virulence-associated accessory chromosome (i.e. chromosome 12) that shows extensive internal duplications, and its induced loss reduces virulence on banana plants, highlighting that fungal genomic diversity and accessory genome variation contribute to pathogenicity (Dijkstra, 2025). In this context, it has been shown that the induced loss of the fusarium accessory chromosome 12 reduces virulence in bananas and is associated with structural reorganizations in central chromosomes. This underscores the importance of this chromosome in pathogenicity and provides new insights into the genomic dynamics of TR4 (Dijkstra et al., 2025).

Due to these factors, there is a significant gap in understanding TR4 resistance in the context of broader banana diversity. Exploring this natural diversity is critical for uncovering and accessing novel resistance sources and enabling breeding strategies for durable resistance. Within this diversity, resistance may result from natural mutations, gene expression dosage, or structural changes such as presence/absence variations, duplications and deletions. For example, induced loss-of-function mutagenesis in TOPLESS family genes (TPL1 and TPL2), associated with susceptibility to *Fusarium* wilt, resulted in a significant reduction in the disease in both tomato and Arabidopsis (Aalders et al., 2024). Exploring this type of strategy in banana, which to our knowledge has not yet been done, could be an option to increase resistance to *Fusarium* wilt (Causier et al., 2012). However, any manipulation of TOPLESS genes would require systematic assessment of potential impacts on growth and yield, since those genes are central transcriptional coregulators and their disruption is likely pleiotropic, potentially affecting multiple developmental and agronomic traits. More broadly, such genetic changes can alter the dosage and regulatory dynamics of defense genes, especially in polyploid genotypes, where gene redundancy and subgenome dominance can modulate response. Additionally, genome constitution differences (e.g., AA, AB, AAA, AAB, ABB) and their genome mosaics (Martin et al., 2023; Sardos et al., 2025) shape the complexity of resistance and must be considered when designing functional analyses. Ultimately, a deeper molecular understanding of the different TR4 defense mechanisms and associated alleles will support modern breeding strategies, including gene editing, that allow precise modifications at specific genomic loci without disrupting other desirable traits (Tripathi et al., 2024; Van den Broeck et al., 2025; Villao et al., 2025),

#### 4.2.3 Conventional breeding

This proposed genotype subset also includes several wild and cultivated diploid banana accessions with high potential in conventional breeding programs. Some, such as ‘Calcutta 4’, ‘Pahang’, ‘Tjau Lagada’, ‘Pisang lilin’ and ‘AAcv Rose’, are well-characterized and widely utilized as parents in existing breeding pipelines due to their resistance traits and fertility, notably for Black Leaf Streak, nematodes and Fusarium wilt Race 1 (Tenkouano et al., 2003; Ndayihanzamaso et al., 2020; Kimunye et al., 2021; Soares et al., 2021; Backiyarani et al., 2024). Several FHIA hybrids, such as FHIA-17 and FHIA-25, which exhibit resistance to Fusarium wilt TR4, have the diploid cultivar ‘Tjau Lagada’ in their pedigree, which is notable through its contribution to the breeding line SH−3217. The same applies to certain NARITA hybrids, which derive their resistance from crosses involving ‘Calcutta 4’, ‘Tjau Lagada’ or ‘Pisang Lilin’ (Ndayihanzamaso et al., 2020). Among these, ‘Calcutta 4’ stands out with the highest quantity of viable pollen, making it a key donor in male-fertile crosses. However, recent studies (Bayo et al., 2024) have highlighted considerable variation in pollen quantity and viability across genotypes in this subset, suggesting a need for systematic screening of wild populations to optimize parental selection. Moreover, several accessions within our proposed subset produce viable pollen (Mukasa and Rubaihayo, 1993; Dumpe and Ortiz, 1996; Rosales et al., 1999) (**Supplementary Table 1**), making them suitable as male parents in breeding programs.

Several lesser-known accessions in the subset may represent valuable untapped resources for developing new breeding populations. For example, *Pisang Cici Alas*, classified as in the wild *Musa acuminata* subspecies, was recently identified as belonging to the *zebrina* subspecies (Martin et al., 2023). It may harbor unique alleles for traits that are poorly represented in domesticated banana genepool, offering an important potential for broadening the genetic base of cultivated bananas (Sardos et al., 2016). Notably, earlier studies have highlighted differences to various races of *Fusarium oxysporum f.* sp. *cubense* susceptibility across *Musa acuminata* subspecies, possibly reflecting local adaptation in Southeast Asia (Vakili, 1965; Buddenhagen, 1986).

#### 4.2.4 Using the banana diversity in intercropping and rotation management

Integrating cultivar diversity and crop rotation practices can substantially mitigate Fusarium wilt incidence in banana production (reviewed in Munhoz et al., 2024). Cultivar mixtures, where resistant and susceptible varieties are grown together, have demonstrated reduced disease prevalence. Already in 1962, Stover (1962) noted moderate disease losses in mixed cultivar and intercrop systems compared to monocultures of susceptible varieties. Similarly, Karangwa et al. (2016) reported a lower Fusarium wilt incidence in farms growing banana cultivar mixtures in landscapes affected by Foc race 1. In these landscapes, East African highland bananas (*Musa* AAA) resistant to race 1 dominate and are cultivated alongside race 1 susceptible ABB and AAB cultivars. Furthermore, recent field observations in Vietnam indicate that reportedly Foc-TR4 susceptible Pisang Awak cultivars, show more limited susceptibility to the disease in mixed-cultivar plantations (Nguyen et al., 2025).

Specific Foc-resistant improved FHIA genotypes (e.g., ‘FHIA01’, ‘FHIA02’), ‘Yunjiao No.1*’* and various GCTCV (‘GCTCV-119’, ‘GCTCV-218’, ‘GCTCV-219’) possess moderate to high Foc TR4 resistance. Their presence reduces Fusarium wilt incidence in field conditions (Molina et al., 2014; Smith et al., 2014; Xu et al., 2018). Furthermore, the combination of resistant cultivars with a practice sustaining soil health influences the rhizosphere microbiome and contributes to a more resilient system by attracting unique beneficial bacterial communities (Troya et al., 2024).

In this context, the wide diversity of resistant cultivars made available through this subset can help strengthen banana-based agricultural systems and make them more resilient in the face of the battle against TR4.

Complementary to cultivar mixtures, intercropping and rotations disrupt disease cycles and enhance beneficial soil bacteria (e.g., *Cyanobacteria*, *Rhodopseudomonas palustris*), which compete with Foc (Fan et al., 2023). Intercropping bananas with antimicrobial plants such as Chinese leek (*Allium tuberosum*) suppresses Foc TR4 by altering soil microbial communities and reducing pathogen viability (Nadarajah et al., 2014; Zuo et al., 2018). Similarly, crop rotation, notably pineapple-banana rotation also results in a significant decline in Foc TR4 populations due to shifts in the soil fungal taxa, with increases in taxa affiliated with *Talaromyces pinophilus* and *Clonostachys rossmaniae* (Yuan et al., 2021). Additionally, cover crops exert allelopathic effects and foster disease-suppressive microbial consortia, further reducing disease pressure (Granzow et al., 2017; Fan et al., 2023). Integration of these strategies into a comprehensive integrated disease management framework including disease-free planting material, and soil amendments could offer sustainable solutions to manage Fusarium wilt of banana.

### 4.3 A scenario for scaling plant multiplication in banana seed systems

#### 4.3.1 Rapid scaling of ITC material for seed systems

**Figure 3:**
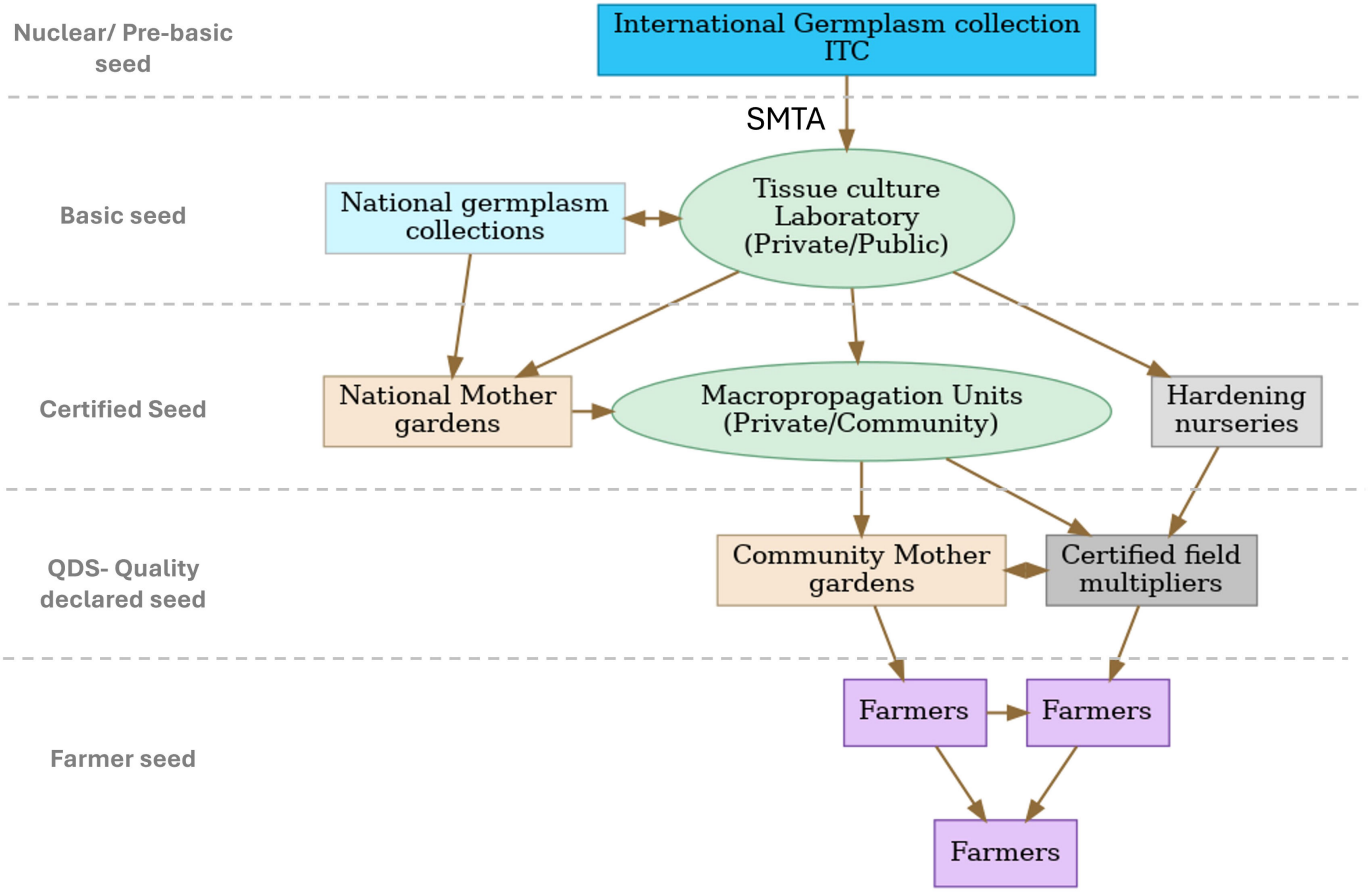
Schematic hub-and-spoke model for multiplication, showing the flow of planting material from international and national collections through production (tissue culture laboratories), quality control, and hardening nurseries to community-level distribution. Arrows represent the direction of germplasm movement and certification oversight, highlighting the roles of NARS research, certification agents, and local farmer networks in maintaining seed quality and traceability.

The International Musa Germplasm Transit Centre (ITC)’s primary role is to conserve, characterize, and distribute clean, true-to-type *Musa* germplasm. However, ITC, as with other clonal species, typically has limited capacity to propagate large numbers of plantlets. Scaling ITC-derived material from a handful of *in vitro* plantlets to thousands of farmer-ready plants requires a robust system for multiplication, quality assurance, and distribution (**Figure 3**). Centralized rapid multiplication laboratories need to be linked to strong distribution infrastructure to ensure timely delivery of planting material. National seed systems can complement this process by guaranteeing access to farmer-preferred cultivars and ensuring availability of high-quality seed in farmer-usable formats (e.g., tissue culture plantlets, macropropagules, and suckers in Africa). The technical, regulatory, and logistical requirements for integrating new material would depend on the cultivar type. Variants of locally cultivated landraces can often be introduced directly, whereas new or improved varieties require multi-site assessment before release. A nice example of a massive introduction of ITC material was made in the late nineties during the Kagera Community Development Program. By July 2002, about 71,000 *in vitro* plants from 25 different genebank accessions had been imported and produced in the Kagera Region in Tanzania (Smale and Tushemereirwe, 2007).

#### 4.3.2 Evaluation of new cultivars

Pest tolerant material received from the ITC will mostly have gone through agronomic evaluation against specific biotic constraints. For new cultivars, germplasm distribution plans must integrate acceptability, and organoleptic trials as formal stages of adoption, performed by national programs. Resistant cultivars need to offer acceptable agronomic performance and consumer and market acceptance, including taste, texture, cooking quality, brewing suitability, and bunch traits. In Uganda and Tanzania, participatory varietal selection (PVS) revealed that although FHIA hybrids showed strong disease resistance, farmer adoption was limited until taste and cooking qualities were validated (Nowakunda et al., 2015). Farmer participation through participatory variety trials ensures new germplasm aligns with socio-economic needs and farming systems. On-farm trials and community mother gardens allow direct evaluation of agronomic traits, disease resistance, yield, and consumer preferences, supporting informed adoption, such as with the Tricot approach (De Sousa et al., 2024). Once new cultivars meet both agronomic and consumer acceptance criteria, they can be integrated into national seed systems and rapidly multiplied through structured dissemination models.

#### 4.3.3 The hub-and-spoke model for multiplication

Several variations of the hub-and-spoke direct multiplication model have proven effective. The hub (a centralized multiplication facility) can receive starter stocks from the ITC under strict phytosanitary conditions and within the framework of a Standard Material Transfer Agreement (SMTA). Hubs are typically state-owned or private tissue culture (TC) laboratories. These facilities ensure varietal identity and health status, producing certified plantlets equivalent to pre-basic seed destined for approved collections and mother gardens. However, receipt of starter stocks by a TC laboratory does not, by itself, authorize direct use due to the restrictions of the SMTA, and may require additional agreements and compliance with national seed laws and certification procedures. Secondary hubs (e.g., accredited macropropagation units) multiply material from these collections under recommended plant hygiene protocols. This supply decentralized institutional or regional mother gardens, which in turn supply certified seed producers. The spokes are decentralized multipliers such as regional nurseries, private enterprises, and farmer cooperatives. Spokes further multiply and distribute planting materials under acceptable hygiene standards, at affordable prices, in line with FAO’s Quality Declared Planting Material (QDPM) standards (Fajardo, 2010). At the community level, farmer-led mother gardens and small nurseries form the final spoke, multiplying low risk planting material under local conditions. Often managed by youth and women, these enterprises can produce farmer-ready planting material through macropropagation or field-based vegetative propagation. The fields also provide an opportunity for direct assessment of the growing plants to support adoption of new varieties. The hub and spoke model supports access to seeds in remote areas, reduces transport costs, and supports local entrepreneurship.

This structure may vary in different countries, depending on the level of seed system regulation, and as such some players may be completely absent. Such gaps could also be filled through farmer exchange of seed only relying on trust perceptions (McEwan et al., 2021; Nduwimana et al., 2022). For example, in Burundi and Malawi, the banana bunchy-top disease (BBTD) control programs, where no local starting material was available, plantlets were produced in private and institutional tissue-culture laboratories, either locally or abroad. These were distributed through community hardening nurseries, which planted materials into mother gardens managed by farmer cooperatives (Nduwimana et al., 2022). From these mother gardens, high-quality seed was then further multiplied and distributed to farmers.

In conclusion, the authors recommend the use of a certified, ready-to-use subset of Foc-TR4-resistant banana genotypes to help ensure more resilient banana-based agro-systems. These ITC-based accessions represent a vital global resource for research, breeding, and sustainable production. Their documented origin ensures traceability, reproducibility, and data integration, making them ideal for scientific and practical applications. These accessions provide a source of Foc-TR4 resistance, at the same time offering untapped agronomic, nutritional, and post-harvest traits. Molecular studies highlight the complexity of resistance mechanisms, while conventional breeding and integrated management strategies show promise for strengthening resilience. However, regional evaluations, long-term field trials, and efficient seed system scaling remain critical to realizing their full potential.

## Supplementary material

Supplementary Table 1 Curated list of banana accessions compiled from published sources, including passport data, genomic classification, and trait-based descriptors. Columns detail the DOI; accession name and code; genome constitution; subspecies/subgroup; country of origin; and genebank availability (e.g., ITC). Additional columns summarize evaluation context such as TR4 (Tropical Race 4) reaction; publication and evaluation year; field verification; and usual use (e.g., Cooking, Dessert, Both, Non-edible). Traits such as pollen and seed fertility and notes on other agronomic characteristics are also provided.

Supplementary Table 2 Concise comparative summary of materials and methods from publications reporting field-based disease evaluations of banana accessions. Includes the year of publication; year and country of evaluation; plant treatment conditions; whether field evaluation was conducted, disease ranking, and meteorological conditions of the evaluation site, if reported.
